# Using graph neural networks for site-of-metabolism prediction and its applications to ranking promiscuous enzymatic products

**DOI:** 10.1093/bioinformatics/btad089

**Published:** 2023-02-15

**Authors:** Vladimir Porokhin, Li-Ping Liu, Soha Hassoun

**Affiliations:** Department of Computer Science, Tufts University, Medford, MA 02155, USA; Department of Computer Science, Tufts University, Medford, MA 02155, USA; Department of Computer Science, Tufts University, Medford, MA 02155, USA; Department of Chemical and Biological Engineering, Tufts University, Medford, MA 02155, USA

## Abstract

**Motivation:**

While traditionally utilized for identifying site-specific metabolic activity within a compound to alter its interaction with a metabolizing enzyme, predicting the site-of-metabolism (SOM) is essential in analyzing the promiscuity of enzymes on substrates. The successful prediction of SOMs and the relevant promiscuous products has a wide range of applications that include creating extended metabolic models (EMMs) that account for enzyme promiscuity and the construction of novel heterologous synthesis pathways. There is therefore a need to develop generalized methods that can predict molecular SOMs for a wide range of metabolizing enzymes.

**Results:**

This article develops a Graph Neural Network (GNN) model for the classification of an atom (or a bond) being an SOM. Our model, GNN-SOM, is trained on enzymatic interactions, available in the KEGG database, that span all enzyme commission numbers. We demonstrate that GNN-SOM consistently outperforms baseline machine learning models, when trained on all enzymes, on Cytochrome P450 (CYP) enzymes, or on non-CYP enzymes. We showcase the utility of GNN-SOM in prioritizing predicted enzymatic products due to enzyme promiscuity for two biological applications: the construction of EMMs and the construction of synthesis pathways.

**Availability and implementation:**

A python implementation of the trained SOM predictor model can be found at https://github.com/HassounLab/GNN-SOM.

**Supplementary information:**

Supplementary data are available at *Bioinformatics* online.

## 1 Introduction

Identifying sites-of-metabolism (SOMs) can significantly enhance our understanding of metabolism. Such sites refer to specific sites within molecules that are susceptible to chemical change. SOMs can therefore be defined as specific atoms (Finkelmann *et al.*, 2018; Kirchmair *et al.*, 2013; Tyzack and Kirchmair, 2019) and/or bonds (Dang *et al.*, 2020; He *et al.*, 2017), and, less conventionally, pairs of unshared valence electrons (Dang *et al.*, 2020). A primary application that has driven the development of SOM prediction tools is determining the enzymatic transformations of xenobiotic compounds including drug molecules. Such transformations are generally classified in two groups—‘Phase I’ activity involving oxidation–reduction and hydrolysis reactions and ‘Phase II’ reactions referring to conjugation transformations (Testa *et al.*, 2012). Cytochrome P450 (CYP) enzymes, a superfamily of structurally diverse metabolic enzymes with broad specificity, are known to be responsible for the metabolism of over 70% of all drugs in use (Zanger and Schwab, 2013; Zaretzki *et al.*, 2013) and are the primary facilitators of ‘Phase I’ reactions (McDonnell and Dang, 2013). As such, CYP enzymes have been the focus of many SOM prediction methods. RS-Predictor (Zaretzki *et al.*, 2012), XenoSite (Zaretzki *et al.*, 2013), Rainbow XenoSite (Dang *et al.*, 2020) and others (He *et al.*, 2017) predict SOMs for specific CYP isoforms.

Non-CYP enzymes, however, play a role in a number of ‘phase I’ interactions and are primary drivers of ‘phase II’ metabolism, e.g. UDP-glucuronosyltransferases (UGT) and sulfotransferases (SULT) (Dixit *et al.*, 2017). Techniques such as FAME (Fast Metabolizer) (Kirchmair *et al.*., 2013) and MetScore (Finkelmann *et al.*, 2018) address this issue by aiming for high prediction accuracy in both ‘phase I’ and ‘phase II’ metabolism. Such approaches therefore are broader than CYP-specific or phase-specific activity prediction tools. Further, there are no prior works that assess the loss in accuracy when broadening activity predictors.

Importantly, broad enzyme specificity is not restricted to CYP enzymes or only those associated with phase I and II metabolism. Most, if not all, enzymes are promiscuous, acting on substrates other than the ones they evolved to catalyze (Nobeli *et al.*, 2009; Tawfik, 2020). Three applications, constructing of de novo synthesis pathways (Otero-Muras and Carbonell, 2021), creating extended metabolic models (EMMs) that account for enzyme promiscuity (Porokhin *et al.*, 2021), and identifying metabolic products measured through metabolomics (Strutz *et al.*, 2022), have driven the development of tools to analyze broad promiscuity. The prevailing approach is to first identify a set of reaction rules (e.g. Duigou *et al.*, 2019; Kotera *et al.*, 2004; Sivakumar *et al.*, 2016) in the form of a local biochemical transformation, followed by matching them to query molecules. Matching rules specify the site of the transformation as well as its local neighborhood to ensure they are sufficiently specific to generate likely promiscuous products. Tuning this specificity (e.g. by radius adjustment) however is a challenge and matching rules may still yield infeasible biotransformations. When paired with rule-based methods, machine learning (ML)-based SOM prediction approaches provide two major advantages. First, they can account for a wider molecular context and learn specialized representations necessary for accurate predictions. Second, they provide a continuous likelihood estimate for the SOM, allowing the ranking of promiscuous products. These improvements can enrich the results of rule-based methods and broaden their applications.

We explore in this work graph-based deep-learning techniques for predicting SOM. Specifically, we use Graph Neural Networks (GNNs) (Zhang *et al.*, 2019; Zhou *et al.*, 2020) (GNNs) to learn atom representations in their molecular graph context in an end-to-end fashion (Donti *et al.*, 2017). These representations can be utilized in downstream classification tasks: either to predict the likelihood of an atom being an SOM or to predict the likelihood of a bond between two atoms being an SOM. A major advantage of using GNNs for SOM prediction is the more natural problem representation as atoms and bonds in molecular structures trivially correspond to nodes and edges in graphs, respectively. Therefore, GNNs can provide more meaningful molecular representations than neural networks used in XenoSite (Zaretzki *et al.*, 2013) and Rainbow XenoSite (Dang *et al.*, 2020), as well as other techniques. Another important advantage of utilizing GNNs over current traditional ML approaches, e.g. Random Forest and Support Vector Machines (SVM), is effective representation learning, which helps avoid the burdensome task of feature selection. In MetScore (Finkelmann *et al.*, 2018), feature combinations were optimized separately for ‘phase I’ and ‘phase II’ interactions. FAME (Kirchmair *et al.*, 2013) considered information gain as their selection criterion for descriptors. XenoSite (Zaretzki *et al.*, 2013) uses a variety of feature types including quantum chemical properties and SMARTCyp (Rydberg *et al.*, 2010) reactivity. He et al. used a set of four feature selection algorithms prior to classification. The need for feature selection is reflective of the large input dimensionality (Saeys *et al.*, 2007) required by those methods. In contrast, the representation learning capabilities of GNNs allow models to perform well with a handful of basic features, such as atom element types and enzyme category labels. We explore several GNN models and show that the GNN-SOM, built using the Chebyshev convolutional operator (Defferrard *et al.*, 2016), outperforms other GNN and traditional ML models when trained to predict the reaction center as defined in the KEGG database (https://www.genome.jp/kegg/reaction/KCF.html). Using labeled reaction centers from the KEGG database(Yamada *et al.*, 2005), we explore several GNN models and show that the GNN-SOM, built using the Chebyshev convolutional operator (Defferrard *et al.*, 2016), outperforms other GNN and traditional ML models (Random Forest, MLP and AdaBoost) that were used in prior works. Further, we demonstrate the utility of SOM prediction for improving rule-based enzyme promiscuity prediction in the context of creating EMMs and constructing synthesis pathways. Our primary contributions are:

- Formulating the SOM prediction problem across all enzyme classes (not just CYP or phase I/II enzymes) as a classification task on either atoms (the atomic SOM problem) or as a classification task on pairs thereof (the bond SOM problem).
- Exploring several GNN models and demonstrating the effectiveness of GNNs in representation learning and SOM prediction over traditional ML approaches that require feature selection.
- Demonstrating the relative difficulty of SOM prediction for CYP-mediated reactions and showing that our general-purpose SOM prediction model performs as well as the versions specific to CYP and non-CYP interactions, thus alleviating the need for separate predictors for the two cases.
- Presenting two biological applications of our SOM predictor, where identifying SOMs measurably improves the quality of predicted promiscuous products and identification of 3-HP synthesis pathways in *Escherichia coli*.

## 2 Methods

### 2.1 Problem formulation: SOM prediction

**Fig. 1.**
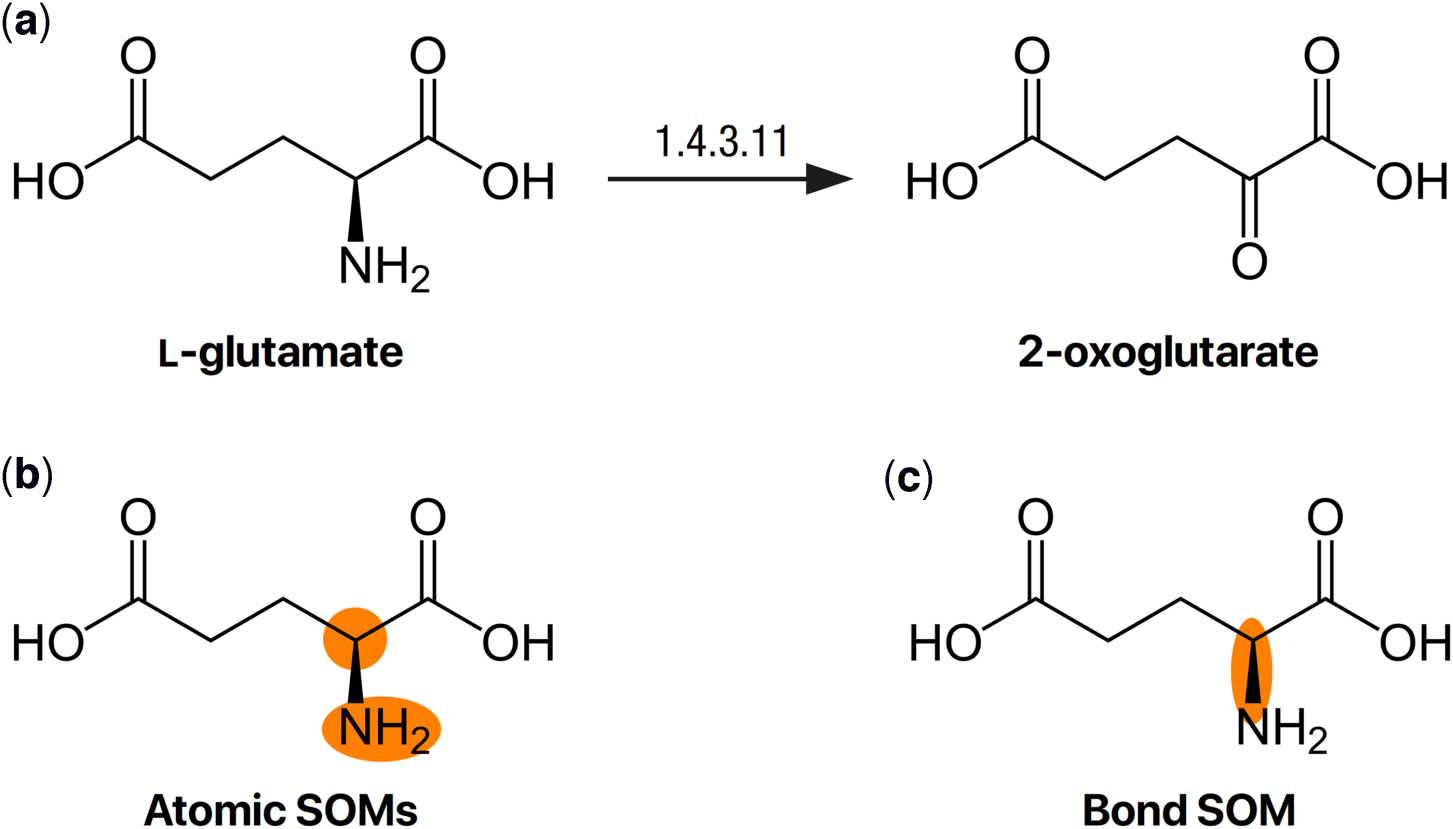
An example of a reaction and the corresponding sites of metabolism. (**a**) In this biotransformation, the enzymatic activity of glutamate-oxidase (EC 1.4.3.11) causes the amine group in l-glutamate (left) to be replaced with a carbonyl group, leading to the formation of 2-oxoglutarate (right). (**b**) On a structural level, the biotransformation converts a single bond to a double bond, in addition to replacing a nitrogen atom with an oxygen atom. As a result, both atoms (oxygen and nitrogen) across the modified bond are considered ‘atomic SOMs.’ (**c**) Alternatively, the modified bond itself could be considered a ‘bond SOM’

As in prior ML methods (Dang *et al.*, 2020; He *et al.*, 2017: Finkelmann *et al.*, 2018; Kirchmair *et al.*, 2013; Zaretzki *et al.*, 2013), the SOM prediction problem can be formulated as a binary classification task. We formally define the ‘atomic SOM’ prediction problem as follows: given a query enzyme and a query molecule, the model predicts the likelihood of each *atom* being an SOM. Similarly, we define the ‘bond SOM’ prediction problem as a bond classification problem, where we predict the likelihood of each *bond* being an SOM. Figure 1 provides an example of atomic and bond SOMs.

Each molecule is represented using an undirected graph *G =* (*V, E*), where every node *i ∈ V* represents an atom and every edge (*i, j*) *∈ E* for nodes *i, j ∈ V* represents a bond. The atomic SOM prediction problem then becomes a *node* classification task. Given a representation **x**_*i*_ for atom *i ∈ V*, we seek to find its predicted SOM label *ŷ_i_*.

We formulate the bond SOM prediction problem similarly to a link prediction task: the objective is to make the determination *ŷ_i,j_* whether a bond SOM exists between a pair of atoms *i*, *j* ∈ *V* with their respective representations **x**_*i*_ and **x**_*j*_. The link prediction is performed by applying a multi-layer perceptron (MLP) to a concatenation of the two atom representations. Since there is no order preference on the atoms, this calculation is performed on both **x**_*i*_ ‖ **x**_*j*_ and **x**_*j*_ ‖ **x**_*i*_ arrangements and the reported prediction is the average of the two. We therefore calculate this prediction as follows:


(1) $${\hat{y}}_{i,j}\;=\;\frac{\mathrm{MLP}(x_{i}\;||\;x_{j})}{2}\;+\;\frac{\mathrm{MLP}(x_{j}\;||\;x_{i})}{2}.$$

### 2.2 Representation learning using GNNs

**Fig. 2.**
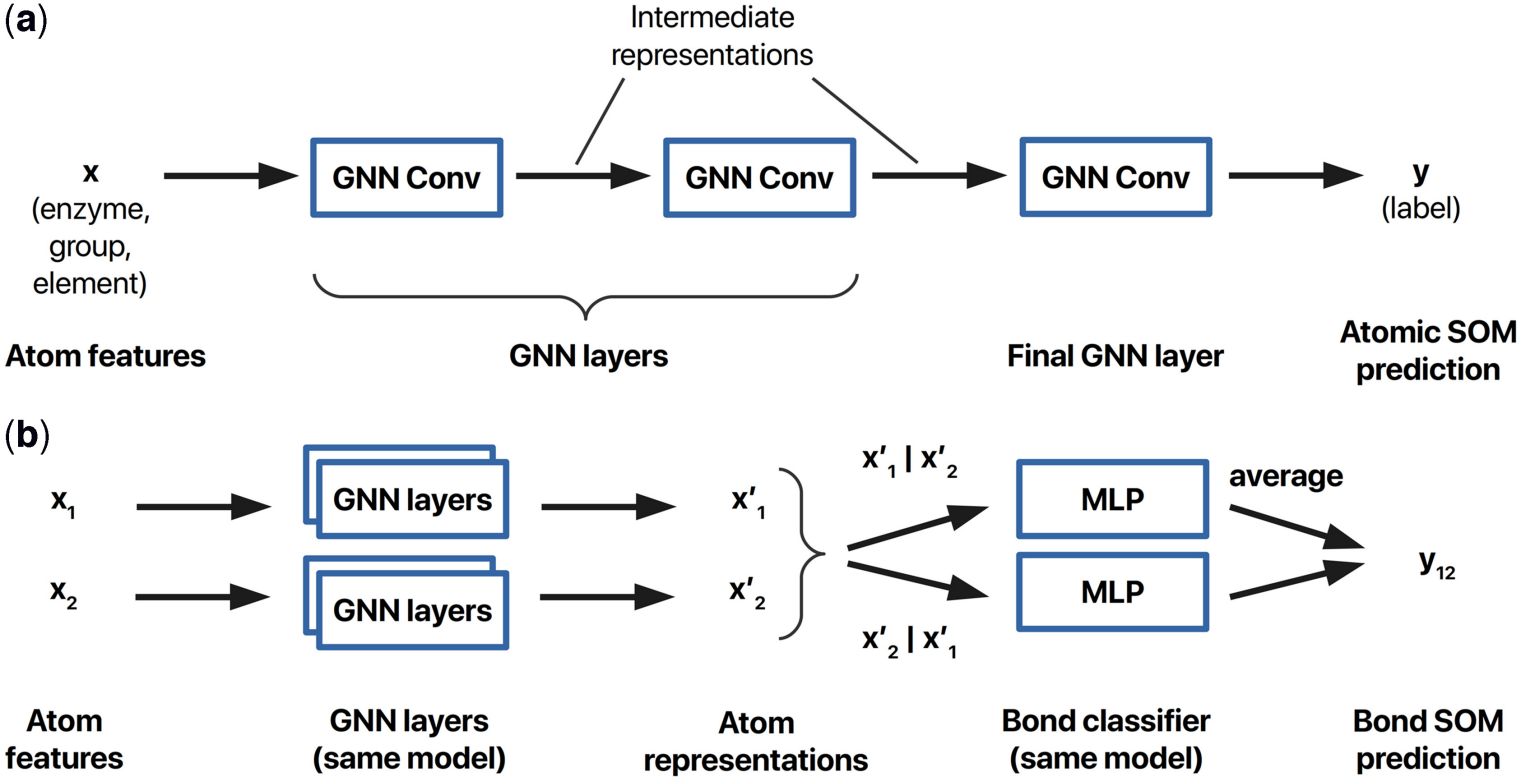
Architecture of GNN-SOM models. (**a**) GNN-SOM for atomic SOM prediction. Atom features **x** are processed through a number of GNN layers, each generating intermediate representations of a fixed size. The last intermediate representation is used as the input to the final GNN layer that generates a single value representing the SOM prediction for that atom. (**b**) GNN-SOM for bond SOM prediction. Atom features for both endpoints of a bond, **x**_1_ and **x**_2_, are processed through the same GNN layers as before; however, the final GNN layer is excluded. The resulting intermediate representations, **x**'_1_ and **x**'_2_, are concatenated two different ways and provided to a bond classifier MLP. The MLP makes two predictions, one for each concatenation, and the average of those represents the final SOM prediction for the bond

For learning node representations, we consider GNNs that consist of a series of layers operating on atom-level embeddings. Such layers can be used to directly make per-atom SOM predictions as in Figure 2a, or using a separate bond classifier MLP for bond SOM predictions as in Figure 2b. Each convolution layer extracts local substructure features for individual nodes and learns a compact representation thereof. We evaluate several GNN message passing layer types for predicting SOMs. The graph convolution operator from Graph Isomorphism Networks (GINs) (Xu *et al.*, 2018) is used as a representative example of a spatial GNN layer. In that work, the authors noted that any aggregation based GNN is at most as powerful as the Weisfeiler–Lehman test and proposed an architecture that generalizes this test. Given input node features **x**, transformed features $x_{\;}^{\prime }\;$are calculated for every node *i* as follows:


(2) $$x_{i}^{\prime }\;=\;\mathrm{MLP}\left((1\;+\;\epsilon )x_{i}\;+\;\sum_{j\;\in N(i)}x_{j}\right),$$

where *ϵ* is a learnable parameter and *N*(*i*) is the set of neighbors of node *i*. In this framework, an MLP is applied to the linear combination of features around the node with the aim of generating unique representations for different neighborhoods.

We also consider a GNN convolution layer designed to mimic circular molecular fingerprints (Duvenaud *et al.*, 2015). Circular fingerprinting identifies important substructures in a molecule and encodes them in a binary vector. The conventional algorithm for generating such fingerprints features several non-differentiable operations on node features, most notably concatenation and hashing. In their GNN rendition of the molecular fingerprinting (MF) GNN concept, the authors replaced the concatenation by a summation over node features; the hashing was replaced by a multiplication of node features by degree-specific learnable weight matrices—one for a node’s own features, **W**, and one for the sum of its neighbors’ features, **H**. For organic molecules, the maximum number of degrees is expected to be five. The resulting convolution is defined as follows:


(3) $$x_{i}^{\prime }\;=\;W^{\left(\deg(i)\right)}x_{i}\;+\;H^{\left(\deg(i)\right)}\;\sum_{j\;\in \;N(i)}x_{j}.$$

The key difference between the MF GNN and GIN is its variability with respect to a node’s degree, or in other words, its neighborhood size. The molecular fingerprinting convolution learns a separate linear mapping of node features to representations for every degree. The GIN approach, on the other hand, learns one mapping for all degrees. With more learnable parameters in the model, MF GNNs can capture richer representations.

The third GNN architecture leverages the Chebyshev convolutional operator, originally proposed by Defferrard *et al.* (2016). Unlike the previous two approaches, this spectral graph method considers a wide portion of a graph at any given GNN layer. It uses a *K*-polynomial filter that integrates features within the *K*-hop neighborhood of a node, which in turn helps it achieve good localization in the node domain (Zhang *et al.*, 2019). Our approach relies on the following convolution definition (Fey and Lenssen, 2019):


(4) $$\begin{array}{l} x_{\;}^{\prime }=\Sigma_{k}{{}_{=1}}^{K}Z\left(k\right)\;\cdot \Theta_{k}\\ Z\left(1\right)=x\\ \begin{array}{l} Z\left(2\right)=\bar{L}\cdot x\\ Z\left(k\right)=2\;\cdot \bar{L}\cdot Z\left(k–\;1\right)\;–Z\left(k–\;2\right) \end{array} \end{array},$$

where the summation above represents the *K*-polynomial filter, **Z**(*k*) is the Chebyshev polynomial of order *k* approximating the spectral graph convolution (Zhang *et al.*, 2019), **Θ** represents the learnable parameters of the filter, $\bar{L}$ refers to the normalized graph Laplacian and *K* is the Chebyshev filter size. The features at the input of the layer are given by **x**. An example showcasing the three convolutional layers is provided in the Supplementary Section S1.4.

For all three GNN models, the node classification task is implemented by inserting an additional graph convolutional layer, accepting **x**_*i*_ as the input and generating *ŷ_i_* as the output, while the edge classification task is implemented using an MLP on the concatenation of two atom representations **x**_*i*_ and **x**_*j*_ as described earlier.

The models are trained using Adam optimization (Kingma and Ba, 2014), with binary cross-entropy as the loss function. Hyperparameters are tuned using the grid search approach. Ranges for the hyperparameters were selected manually based on their expected effects and practical considerations for runtime. The hyperparameters include the size of the latent representations (set to 64, 128, 256 or 512) and the number of GNN layers (ranging from 1 to 5). In addition, we adjusted the filter size (from 1 to 10) for GNNs for the Chebyshev convolutional operator, and maximum number of degrees (from 1 to 5) for models using the molecular fingerprinting convolution. The models were constructed using the convolutional layer implementations provided by PyTorch Geometric (Fey and Lenssen, 2019).

### 2.3 Baseline models

Random Forest, MLP and AdaBoost are selected as baseline models for the same classification task. These models have been used prior for SOM prediction in on reaction-specific datasets and can be considered representative examples of the current state of the art in SOM prediction. For example, Random Forest (RF) classifiers form the basis of the MetScore (Finkelmann *et al.*, 2018) and FAME (Kirchmair et al., 2013) methods. XenoSite (Zaretzki *et al.*, 2013) and Rainbow XenoSite (Dang *et al.*, 2020) utilize MLP models for their prediction task. Finally, the work of He et al. (He *et al.*, 2017) leverages a collection of ML techniques, including RF and AdaBoost. We use the same input feature vectors and datasets for both our GNN and baseline models, therefore allowing for a fair comparison between baselines and GNN models.

The MLP models are trained using Adam optimization with cross-entropy loss. For Random Forest models, we used Gini impurity as the splitting algorithm. The SAMME.R boosting algorithm was used in building the AdaBoost models. The models were constructed using the scikit-learn package. Hyperparameter tuning is performed using grid search. For MLP, we varied the size of the latent representations (set to 32, 64, 128, 256 or 512) and the number of hidden layers (from 1 to 5). Meanwhile, for both Random Forest and AdaBoost, we adjusted the number of decision trees (set to 100, 250, 500 or 1000).

### 2.4 Dataset construction

We derive our atom SOM dataset from the KEGG RPAIR database (Yamada *et al.*, 2005), a collection of atom-mapped reactant pairs associated with specific transformation patterns. Each RPAIR has cross-references to reactions in the KEGG database as well as the relevant Enzyme Commission (EC) numbers. The transformation patterns are encoded using R, D and M (RDM, for short) atom-level tags. ‘R’ distinguishes the reaction center atoms, which we assume to be the sites of metabolism. The ‘D’ (difference) tag points to molecular substructures modified by the biochemical transformation, while the ‘M’ (match) tag refers to substructures neighboring the ‘R’ tagged atom that remain unchanged.

We create separate versions of the dataset, one for the atomic-SOM problem, and one for the bond-SOM problem. To create the atom-centric version of the dataset we assign SOM labels to every atom at the reaction center of an RDM pattern (Supplementary Section S1.1). To create a dataset suited for bond SOM prediction, we identify bonds that change due to a chemical transformation and label them as SOMs instead (Supplementary Section S1.2). As this dataset is not readily available, we curate the KEGG RPAIR database to generate such a set. The dataset was further processed to improve SOM labeling (Supplementary Section S1.3). Some adjustments are specific to a given version of the dataset. A symmetry adjustment is applied to both datasets to account for molecular symmetrical features.

The feature vectors representing atoms consist of three components, which include the atom elemental type, the KEGG atom type (an atom label that represents the atom type and the atom’s relationships to nearby elements and bonds), and the first two levels of the EC (Enzyme Commission) number of the enzyme associated with the transformation. Further details can be found in Supplementary Section S1.

## 3 Results

### 3.1 Data splits

To assess the effectiveness of the models on the available dataset (21 023 molecules), we employ a cross-validation scheme. We create ten different data splits, with 80% of molecules allocated for training, 10% for validation, and the remaining 10% for testing. The partitions are generated by performing shuffle splits on the set of biotransformations, which ensures there is no information leakage across the substrate and product sides of a reaction or across enzymes associated with the same transformation. The set of training molecules is used to train several versions of a model with different hyperparameter combinations. The best combination is then selected based on its performance on the validation set. Finally, the selected model is evaluated on the test set. This process is repeated ten times, once for each data split, and we report the average test set performance for each method.

This approach yields ten different models with varying training set molecules and hyperparameters. For use in biological applications, we create a combined model that applies all ten models to a query molecule and reports the average predicted SOM probabilities for each atom.

### 3.2 GNN model evaluation

In our evaluation of various models, we considered several performance metrics, including area under the ROC curve (AUROC) and *R*-precision. AUROC evaluates the model’s ability to rank SOM sites higher than any site where metabolism is not known to occur. *R*-precision measures the fraction of SOMs present among the top *R* predictions, where *R* is the total number of SOMs in the molecule or the test set.

We calculated AUROC and *R*-precision using two different methods. The ‘molecular’ benchmarks were defined to be the average value of the corresponding metric when calculated on a per-molecule basis. This represents the expected performance in applications where the objective is to identify SOMs in a specific queried molecule. The alternative approach is to calculate AUROC and *R*-precision on the full set of atoms where all molecules are pooled together. Such ‘atomic’ measurements are representative of a model’s performance in scenarios where the goal is to locate the most likely SOMs in a group of molecules. We also considered the ‘top-two’ correctness rate, a molecular metric that is frequently used to evaluate the performance of CYP SOM predictors (Dang *et al.*, 2020; Zaretzki *et al.*, 2013). It represents the proportion of molecules where a known SOM appears among the molecule’s top-two predicted sites. Therefore, in contrast to the other measures, it ‘saturates’ and does not require a completely correct ranking for a perfect score to be assigned to a molecule. Several versions of each model with different hyperparameters were evaluated. Although there were five performance metrics that we considered important, they were all strongly correlated with one another. As such, model selection was performed by maximizing the molecular *R*-precision on the validation set.

Model evaluation is reported for the best-in-class GNNs (Table 1). GNNs using the Chebyshev convolutional operator achieved the best performance by all five metrics, with models based on the molecular fingerprinting convolution being a close second. Performance of GIN-based models, however, was markedly worse. For the remainder of the manuscript, we refer to the GNN that utilizes the Chebyshev convolutional operator as GNN-SOM.

**Table 1.** GNN and non-GNN model evaluation

	Molecular R-precision	Molecular AUROC	Top-2 correctness rate	Atomic R-precision	Atomic AUROC
(A) GNN model evaluation					
GNN-SOM (GIN)	0.676	0.915	0.812	0.635	0.939
GNN-SOM (MF)	0.764	0.946	0.851	0.731	0.963
GNN-SOM (Cheb)	0.789	0.953	0.868	0.771	0.971
(B) Models with KEGG atom types					
RF	0.520	0.850	0.716	0.469	0.872
Ada	0.520	0.850	0.713	0.470	0.871
MLP	0.519	0.850	0.714	0.470	0.871
GNN-SOM	0.789	0.953	0.868	0.771	0.971
(C) Models without KEGG atom types					
RF	0.294	0.649	0.501	0.276	0.713
Ada	0.295	0.650	0.502	0.276	0.713
MLP	0.297	0.651	0.505	0.275	0.711
GNN-SOM	0.749	0.934	0.849	0.725	0.963
(D) Expectation via random guessing					
Expectation	0.111	0.500	0.111	0.044	0.500

*Note*: (A) Different GNN models. (B) Performance on the original dataset that includes the KEGG atom types (C) Model performance with the removal of the KEGG atom types. (D) Expected performance that would be achieved on this dataset via random guessing. Standard deviation for all listed values is 0.02 or less.

### 3.3 GNN models outperform non-GNN models

We compared the best GNN model, GNN-SOM, to several baseline approaches. GNN-SOM outperforms baseline methods on all five considered metrics (Table 1). The use of GNNs led to an approximately 10% improvement in molecular and atomic AUROCs and as much as a 30% increase in atomic *R*-precision.

We postulate that the limited information about an atom’s local chemical environment is a major factor responsible for performance differences. To evaluate the contributions of the KEGG atom types, we removed the node feature responsible for this property and observed a 15–20% reduction of the baseline models’ evaluation metrics, while the performance of GNN-based models was only minorly affected. Baseline models are able to utilize the KEGG atom types as a part of the node feature vector, while GNNs can natively infer it from the graph structure.

We provide the expected performance achievable via random guessing as a means of evaluating the inherent complexity of the prediction problem. A binary classifier making random guesses with no bias towards any specific class achieves AUROC of 0.5 (Fawcett, 2006). Given *R* relevant SOMs and *N* total SOM candidates, the expected *R*-precision and top-two correctness rates for a set of atoms were calculated as follows:


(5) $$\mathrm{Rprecision}\;=\;\frac{1}{R}\sum_{i\;=\;0}^{R\;-\;1}\frac{R-\;i}{N\;-\;i},$$

$$\mathrm{Top}\mathrm{Two}\;=\;\frac{1}{2}\sum_{i\;=\;0}^{1}\frac{R\;-\;i}{N\;-\;i}.$$

In each iteration of a summation, we computed the expected number of selected SOMs from the set of the remaining available candidates. For *R*-precision, we considered the total number of sites selected among the top *R* prediction attempts and divided it by the number of attempts made, following the definition of the metric. For top-two correctness rate, we found the probability of selecting an SOM within the first two attempts using a similar approach.

### 3.4 Bond SOM prediction and atomic SOM prediction

We found that the bond SOM prediction problem is inherently more difficult than the atomic SOM prediction, with limited advantages offered in the way of combining the two approaches. The *expected* performance metrics, with the exception of AUROC, are lower for the bond SOM prediction task by approximately a factor of two: this is a consequence of there being about twice as many SOM edge candidates in the bond prediction problem with no proportional increase in the number of true sites. To quantify the potential benefits of combining the two problems for more accurate predictions overall, we compared molecular *R*-precision values achieved by the best models for each data split, counting the number of molecules where the performance of one model exceeded that of the other or where there was a tie. On average across the ten data splits, the node-centric model achieves better molecular *R*-precision for 733.3 molecules (SD 40.6), the edge-centric model outperforms in 172.3 (SD 25.4) cases and the remaining 1202 (SD 46.0) molecules experienced identical performance on the two models. Therefore, in most cases, the node-centric model would be the ideal choice, with it providing better predictions for a larger number of molecules. The comparison of the overall performance of the two types of models is provided in Table 2.

**Table 2.** Performance of GNN-based models on node-centric and edge-centric versions of the dataset

	Molecular R-precision	Molecular AUROC	Top-2 correctness rate	Atomic R-precision	Atomic AUROC
GNN-SOM	0.789	0.953	0.868	0.771	0.971
Expectation, atom	0.111	0.500	0.111	0.044	0.500
GNN-SOM-Bond	0.612	0.940	0.777	0.543	0.946
Expectation, bond	0.059	0.500	0.062	0.024	0.500

*Note*: Standard deviations are 0.02 or less.

### 3.5 CYP versus non-CYP prediction

To evaluate the applicability of our model across different types of reactions, we investigated performance of our models on interactions mediated by CYP and non-CYP enzymes. We found that CYP-specific reactions are more challenging than non-CYP interactions for all models. When trained and tested on one type of reaction, GNNs and baseline methods alike performed significantly better on non-CYP interactions despite the expected performance of a random classifier being similar in both sets (Table 3A and B). Furthermore, the models specific to non-CYP reactions—unencumbered by the more challenging CYP interactions—demonstrated slightly better performance compared to general models (Table 2) on their respective molecule sets. We believe this difference was in part due to a comparatively small number of CYP reactions available for training: in our dataset, there were 18 139 non-CYP reactions and 2877 CYP-associated reactions. However, the performance gap between the CYP and non-CYP reactions was less pronounced for our GNN approach compared to the baseline ML techniques, indicating that GNNs can more effectively learn a wide range of SOMs when training data is limited. As before, the GNNs consistently outperformed the baseline models given the same circumstances.

**Table 3.** Performance of GNNs and baseline models on CYP and non-CYP mediated interactions separately

	Molecular R-precision	Molecular AUROC	Top-2 correctness rate	Atomic R-precision	Atomic AUROC
(A) Models trained and tested on only CYP reactions					
RF, CYP	0.359	0.723	0.573	0.359	0.758
Ada, CYP	0.358	0.725	0.575	0.360	0.761
MLP, CYP	0.362	0.728	0.575	0.358	0.758
GNN-SOM, CYP	0.693	0.912	0.767	0.695	0.955
Expectation, CYP	0.108	0.500	0.112	0.051	0.500
(B) Models trained and tested on only non-CYP reactions					
RF, non-CYP	0.545	0.870	0.731	0.488	0.885
Ada, non-CYP	0.546	0.870	0.734	0.489	0.885
MLP, non-CYP	0.547	0.870	0.737	0.488	0.886
GNN-SOM, Non-CYP	0.799	0.958	0.878	0.782	0.973
Expectation, non-CYP	0.111	0.500	0.111	0.044	0.500
(C) Models trained on all reactions but tested only on CYP or non-CYP					
GNN-SOM, CYP	0.690	0.910	0.775	0.694	0.956
GNN-SOM, non-CYP	0.804	0.959	0.883	0.784	0.973

*Note*: Standard deviations are 0.03 or less.

We also found that our general-purpose SOM prediction model is as effective as the CYP and non-CYP-specific models. The SOM predictor trained on all available interactions, achieves very similar performance on the CYP and non-CYP subsets compared to models trained on each reaction type specifically (Table 3C). As such, our approach alleviates the necessity for separate predictors for CYP and non-CYP interactions.

### 3.6 Applications of using SOM prediction

While several techniques predict overall reaction feasibility, such as thermodynamic feasibility [e.g. eQuilibrator (Beber *et al.*, 2022; Noor *et al.*, 2013)], or likelihood of a biochemical conversion between a substrate and a product [e.g. DeepRFC (Kim *et al.*, 2021) and ELP (Jiang *et al.*, 2021)], SOM prediction determines the likelihood of an enzyme class acting on a particular atom or bond within a molecule. Therefore, when paired with a rule-based method, the SOM likelihood can be assumed a proxy for the likelihood of reaction occurrence. We demonstrate the utility of SOM prediction using two applications: screening promiscuous products generated using rule-based prediction methods, and ranking synthesis pathways based on SOM likelihood of each pathway’s individual reaction steps. While we selected PROXIMAL (Yousofshahi *et al.*, 2015) as our companion rule-based method, our GNN-SOM method is independent of PROXIMAL, and can be used with other rule-based techniques as well.

#### 3.6.1 Screening promiscuous products generated from rule-based prediction methods to create EMMs

To evaluate GNN-SOM’s utility in improving prediction of products arising due to enzyme promiscuity, we leveraged GNN-SOM as a screening step for rule-based product prediction methods to eliminate unlikely promiscuous products. We chose to evaluate the impact of including this step on creating EMMs (Amin *et al.*, 2019), which are intended to account for promiscuous enzymatic activities. In that work, the *i*ML1515 model of *E.coli* (Monk *et al.*, 2017) was extended with a number of reactions that could arise due to uncatalogued promiscuous enzyme activity. The PROXIMAL tool was used to predict interactions occurring between native enzymes and substrates listed in *i*ML1515. The resulting putative products were then searched in ECMDB (Guo *et al.*, 2013; Sajed *et al.*, 2016) and PubChem to identify promiscuous transformations that were observed previously but are missing from the *i*ML1515 model. Balanced reactions were then constructed for each transformation, and after manual curation, 23 new reactions were recommended for addition into the model.

PROXIMAL generates products via application of RDM patterns on molecular graphs of queried substrates: it does not consider SOMs. As a result, it may propose unfeasible or unlikely biotransformations. Using SOM predictions as a criterion for applying patterns can help lower the number of such unfeasible products, potentially reducing the amount of manual curation required. We use our SOM predictor as a filter on the products. For a biotransformation to be accepted, the reaction center in the substrate at which PROXIMAL applied the pattern must be at or above a certain threshold. If the reaction center is below that value, we consider the transformation to be unlikely and discard it.

Our application of 1875 PROXIMAL operators presented by Amin *et al.* to 106 high-concentration metabolites (Tepper *et al.*, 2013) in *E.coli* yielded a set of 1989 products, 1390 of which we could cross-reference between the PubChem online REST API and the derivative products presented in the publication. Additionally, there were 55 products that were cross-referenced to ECMDB. Such products were considered to be ‘verifiable’ since there was concrete evidence of their existence in *E.coli*. In comparison, predicted products merely found in PubChem are less likely to occur in *E.coli*, since this set includes a great many metabolites never observed in the organism before. As such, the ratio between the number of compounds confirmed by both databases and PubChem only, represents a metric of interest. Thus, retaining a higher percentage of ECMDB-confirmed products when filtering using GNN-SOM compared to the overall predicted products showcases the utility of GNN-SOM.

**Fig. 3.**
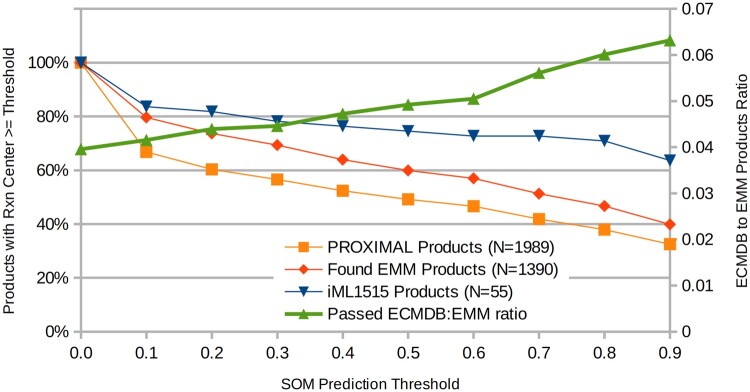
Percentage of products passed by the SOM predictor filter for different sets of metabolites, at different thresholds

Instead of examining the number of products under a fixed threshold (likelihood), we explore how the threshold impacts the number of products. We applied a range of filter thresholds from 0.0 to 1.0 in 0.1 increments (Fig. 3). As the threshold increases, fewer products—confirmed and overall—pass the filter. Importantly, the proportion of confirmed products grows with the threshold. The ratio of verifiable products steadily increases as the threshold is raised (Fig. 3), indicating that metabolites confirmed to exist in *E.coli* are removed at a lower rate than unconfirmed metabolites; the ratio is at its lowest prior to the application of the filter (threshold = 0.0). Therefore, GNN-SOM enables more efficient prediction of metabolites suitable for creating EMMs. While we applied GNN-SOM post-promiscuous product generation as a screening tool, GNN-SOM can also be used to identify likely SOMs where the rules can be applied.

#### 3.6.2 Ranking synthesis pathways based on SOM likelihood

SOM predictors can guide synthesis pathway construction. In this experiment, we compare known engineered two- and three-step pathways for 3-hydroxypropionic acid (3-HP) synthesis in *E.coli* to a number of putative synthesis pathways generated by PROXIMAL. To compare pathways based on SOM-GNN guidance, we calculate a pathway likelihood by taking the product of GNN-SOM predictions for the constituent reactions of each pathway. The output of GNN-SOM is a continuous value, representing a given site’s likelihood of being an SOM for a certain EC number. Since the site and the type of biotransformation is specified, such likelihood is reflective of the probability of occurrence for the entire reaction. The overall likelihood of a pathway is calculated as the product of the probabilities of its reaction steps. Pathway likelihoods thus provide a relative metric that allows comparing pathways based on SOM likelihood. As pathway construction (without filtering, e.g. based on yield or other metrics) generates a large number of putative pathways, we expect engineered pathways to have, on average, a higher pathway likelihood than the putative pathways.

We considered two engineered pathways for 3-HP synthesis known from the literature. The first pathway is catalyzed by a non-native enzyme, malonyl-CoA reductase, to produce 3-HP in two reaction steps (Cheng *et al.*, 2016; Rathnasingh *et al.*, 2012). The second pathway relies on heterologous expression of several genes—*panD*, *gabT* and *ydfG—*to yield 3-HP in three reaction steps starting from L-aspartate (Wang *et al.*, 2014). Both pathways have been successfully used as a part of longer pathways for sourcing 3-HP and derivatives from glucose consumed by *E.coli*. In many reactions comprising those pathways, there were multiple options for the selection of a reaction center. To identify the most plausible set of centers, we calculated GNN-SOM likelihoods for all possible combinations and selected the one that maximized the likelihood of the pathway.

The family of generated pathways was constructed via prediction of promiscuous activity involving 3-HP and enzymes natively present in *E.coli—*and since the bacterium does not natively produce this molecule, such interactions are representative of unsuccessful pathway synthesis outcomes. Working back from the target metabolite, PROXIMAL was used to propose precursor metabolites and the necessary reaction steps (enzyme as well as the reaction center) that could ultimately yield 3-HP. Because the number of such pathways can be extremely large, 50 000 pathways of each depth were sampled at random, followed by removing duplicates. This process produced 7869 two-step and 45 622 three-step synthetic pathways. This set was further refined by removing pathways with intermediates not listed in the PubChem—such intermediates may less likely occur in nature than metabolites previously observed and catalogued in the database. In the end, we obtained 1226 two-step and 1118 three-step PubChem-only pathways.

The average likelihood of putative pathways was 0.24 for two-step and 0.17 for three-step interactions. For PubChem-only synthetic pathways, the mean likelihoods were 0.30 and 0.37 for two- and three-step pathways, respectively—the higher likelihood observed in this case suggests the model assigns greater confidence to interactions with evidence of the metabolite’s existence. Finally, the likelihoods of the two effective synthesis pathways for the most plausible sets of reaction centers were found to be 0.987 and 0.956 in the cases of malonyl-CoA and l-aspartate pathways, respectively.

The calculated likelihoods allow ranking synthetic pathways based on the likelihood of the SOMs they depend on, and ideally, such ordering would prioritize functional pathways over the putative ones. The quality of this ranking can be quantified using the AUROC metric. The likelihood of the two-step malonyl-CoA pathway exceeded those of *all* considered two-step putative pathways, leading to the AUROC of 1.0. For the l-aspartate pathway and its putative three-step counterparts, the AUROC was 0.997. Therefore, our SOM predictor allows unlikely candidates to be deprioritized or removed from consideration at a low computational expense.

## 4 Conclusion

We explored in this article GNN-based models that predict atomic and bond SOMs for enzymatic reactions. Our analysis revealed that the bond-SOM prediction problem is more difficult than the atom-SOM prediction problem. Our GNN model, GNN-SOM, based on the Chebyshev convolutional operator consistently outperforms baseline ML classification models. Importantly, we showed that training on all enzymatic reactions outperforms the same model when trained on only CYP enzymes. Thus, the SOM prediction task (for CYP and non-CYP enzymes) benefits from a larger and more diverse training dataset. We also showed that the use of GNN-SOM can provide ranking on promiscuous products when evaluating the construction of EMMs and synthesis pathways for 3-HP.

There are several avenues for improving our work. Although bond-based GNN models showed lower performance compared to atom-based ones, there were still several molecules where using them would be advantageous. Identifying characteristics of those molecules and combining the two types of models may provide an opportunity to enhance SOM prediction. Adding physicochemical molecule descriptors can improve prediction results as GNN-SOM only considers element and bond information that can be elucidated from the 2D molecular structure. Finally, pre-training the model using a larger molecular dataset could further improve accuracy and applicability of GNN-SOM.

## Supplementary Material

btad089_Supplementary_Data

## Data Availability

The RPAIR data set was downloaded from the Kyoto Encyclopedia of Genes and Genomes (KEGG) database under an academic license.
