# Supplementary material for: Using graph neural networks for site-of-metabolism prediction and its applications to ranking promiscuous enzymatic products
Source: Bioinformatics. 2023 Feb 15;39(3):btad089. doi: 10.1093/bioinformatics/btad089 (PMC9991054; doi:10.1093/bioinformatics/btad089)
Supplement: btad089_Supplementary_Data [file btad089_supplementary_data.pdf]

Using Graph Neural Networks for Site-of-Metabolism Prediction and its Applications to Ranking Promiscuous Enzymatic Products

Supplementary Material

Vladimir Porokhin[1], Li-Ping Liu[1] and Soha Hassoun[1,2]

[1]Department of Computer Science, Tufts University, 177 College Avenue, Medford MA, 02155, USA

[2]Department of Chemical and Biological Engineering, Tufts University, 4 Colby Street, Medford MA, 02155, USA

1 Datasets

1.1 Dataset with Atom SOMs

Node features consist of three components: the KEGG atom type, the atom elemental type, and the Enzyme Commission (EC) number corresponding to the metabolizing enzyme. The KEGG atom type represents the atom's immediate chemical environment in the form of a single label. These types are defined by a set of 68 small-scale molecular substructure patterns (Kanehisa Laboratories), which we further extend to distinguish between atoms connected by single and double bonds (e.g. oxygen and hydroxy group in carboxylic acid). Although there are separate KEGG atom types for different elements, this semantic structure is not conveyed by their feature representation. Therefore, we include a node feature to describe the atom's element separately. The EC number is also incorporated as a node feature. The EC classification system provides a hierarchy of four levels, allowing enzyme behaviors to be represented to various degrees of specificity. Highly specific levels provide more information about the metabolizing enzyme, albeit with the disadvantage of an increased feature complexity and a diminished number of training examples per category. As a compromise, we use the first two levels of the EC system. All three components of the feature vector correspond to separate categorical labels: as such, they're represented using a one-hot encoding scheme.

The use of KEGG atom types allows for streamlined models with wide applicability. Bond properties such as order, strength, and type are all implicit to various extent in the functional group labeling provided by the types, therefore there is no need to encode and process edge features. Furthermore, because the KEGG atom types are reproducible with publicly available definitions (Kanehisa Laboratories) and implementations such as KCF Convoy (Kotera et al.; Sato et al.), models developed here can be easily applied to non-KEGG datasets.

1.2 Dataset with Bond SOMs

Many reactions result in the creation of new bonds that can occur between existing atoms or atoms not originally present in the molecule, for example in ring closure reactions and additions of new functional groups, respectively. To allow these bonds to be considered as potential SOMs, each atom in a molecule is paired with a unique synthetic placeholder node such that the edge between the two represents bonds that may be created at that location. Labeling of bond changes is then done in three stages. First, an atom-to-atom mapping between the substrate and the product of an RPAIR is produced. This mapping is created based on the molecular alignment information provided with the RPAIR database with a modification described below. Any difference-tagged atoms in one of the molecules in an RPAIR are mapped to placeholders in the other molecule. Since there may be multiple such atoms and the mapping is one-to-one, we initially create four placeholders at every atom – a number sufficient to map atoms in every existing RPAIR.

The next step involves checking atoms and bonds aligned by this mapping for changes. We consider three types of differences as indicators of bond SOMs: 1) changes of element at one of the endpoints of a bond, including a change to or from a placeholder, 2) changes in the bond order, and 3) differences in the formal charge of an atom. The latter is encoded as a change in one of the placeholder nodes, which in this case represents a proton or an electron that's being added or removed. Any bonds where such changes occur are labeled as the SOM bonds.

The four placeholder nodes situated at a given atom are indistinguishable from one another: the additional copies were created only to facilitate a one-to-one mapping and are otherwise not needed. The last step, therefore, entails combining the placeholder nodes such that only one remains per atom. The SOM bond labels associated with the placeholder nodes are merged so that if one of the duplicates is marked as a positive SOM, the final merged bond label would be marked the same, too. As a result, all SOMs detected in the previous stage are preserved in the final molecules.

1.3 Dataset Adjustments to Improve SOM Labelling

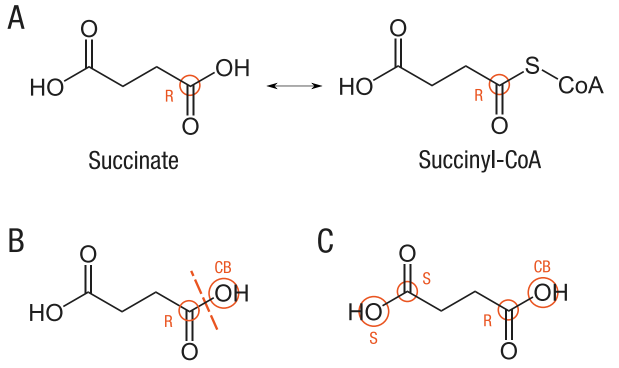

Supplementary Figure 1. Adjustments for cleaved bonds and symmetry demonstrated by a reaction converting between Succinate and Succinyl-CoA. (A) A biotransformation with the reaction center labeled as "R", where the hydroxy group is removed and replaced by a sulfur atom. The hydroxy group in Succinate and the sulfur atom in Succinyl-CoA could be considered as SOMs. (B) The cleaved bond in Succinate and the additional atomic SOM, which is labeled as "CB" (for "cleaved bond"). (C) Two additional atomic SOMs, labeled as "S" (for "symmetry") that are implied by the symmetrical structure of the molecule.

Node-centric dataset adjustments. The node-centric dataset for the atomic-SOM problem is corrected for cleaved bonds. In cases when a bond is broken in a molecule, often only one of the endpoint atoms are marked as the reaction center of the transformation pattern, with no apparent consistency as to which atom that would be. However, both atoms should be considered as the SOM, as they are located at the site of the transformation and are involved to an equal extent. To this end, we track bonds that are cleaved over the course of the reaction, and if one of the end points of such a bond is marked as the reaction center, we make sure to mark the other one as well. Supplementary Figure 1 shows an example reaction where a cleaved bond results in the creation of additional SOMs.

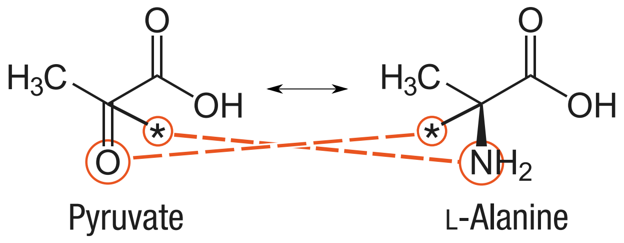

Supplementary Figure 2. Interchange adjustment as demonstrated by a reaction converting between Pyruvate and L-Alanine. In this case, the biotransformation is encoded such that the oxygen atom is removed in Pyruvate and the amino group is added in L-Alanine. Because no direct correspondence is provided between the two, normally each would be mapped to a placeholder atom (only relevant ones shown), resulting in two bond SOMs detected per molecule. However, an equivalent transformation could be obtained by replacing the oxygen atom with the amino group directly, in which case only one bond per molecule would be considered as SOM. Our method identifies such instances and updates the atom mapping to avoid generating spurious SOM labels.

Edge-centric dataset adjustments. The edge-labeled SOM dataset uses molecular alignment information derived from the KEGG database. To support our modified KEGG atom types with bond order suffixes, we introduce a preprocessing step to ensure that the molecular alignment has the appropriate atom mapping. The molecular pairs are scanned, searching for mappings with the modified atom types. The mapping is checked to verify the modified types around a common neighbor are mapped without requiring changes in the bond order suffix. If such case is detected, the mapping is reversed, resulting in an accurate alignment of the modified atom types, consistent throughout the dataset.

Another modification of the alignment information is performed with the goal of simplifying the mapping. In some molecules, the replacement of an atom was recorded as the removal of one substructure and the addition of another with no mapping in between, as opposed to a direct replacement. Despite being an identical operation, the former would produce additional SOMs: because one atom on each side of the reaction has no mapped counterpart on the other, each would be mapped to a placeholder node, resulting in placeholder bonds being marked as the positive SOMs as well. In both cases, however, the bonds with the replaced atom would be marked on both the substrate and the product of the reaction. To ensure such atom replacements are consistently handled as replacements with the simplest possible mapping, we identify those cases and correct them accordingly. Supplementary Figure 2 shows an example reaction where this modification would apply.

Symmetry adjustments. Once all SOMs are selected, the final adjustment entails correction for symmetry. Many molecules have symmetrical features, where multiple sites are structurally identical given the context of the molecular graph. Such features may be as large as half of the entire molecule (e.g., Bisphenol A) or as small as one atom (oxygen atoms in the outermost phosphate group of ATP and other molecules). Equivalent sites are indistinguishable from one another and therefore, should have the same SOM classification. Yet only some of the symmetrical sites may be marked as the SOMs based on the genericized transformation pattern or the available alignment information, providing conflicting information during training or evaluation stages of model design. This issue was previously recognized and handled in MetScore (Finkelmann et al.), but no detailed procedure for this adjustment was provided.

To address the SOM symmetry issues in the dataset, we include a post-processing step that mirrors labeled reaction centers to all symmetric locations as follows. Given a molecular graph, we first assign vertex colors as a function of the KEGG atom type augmented with the bond order suffix. This operation distinguishes atoms by their elements as well as the types of bonds around them. Then, we compute all automorphism groups of the molecular graph – permutations of same-colored atoms that keep the set of bonds exactly the same – using the nauty program (McKay and Piperno). While graph isomorphism is not solvable in polynomial time in the general case, the molecules we consider do not pose a significant computational challenge: the calculation of automorphism groups for all molecules in practice takes only a few minutes. Furthermore, most organic molecules are planar graphs (Rücker and Meringer), for which there exists a linear time graph isomorphism algorithm (Torán and Wagner). Finally, we check the sets of vertices that are equivalent by some automorphism for reaction center labels. If such a set contains a labeled SOM, we consider all vertices in the set to be SOMs also.

This symmetry adjustment is applied to both node and edge versions of the dataset. In case of the edge-centric set, the same algorithm is applied to the line graph of the molecule, where every bond is converted to a line graph node. Associations between bonds are preserved by creating line edges for every pair of bonds sharing a common atom. The line graph nodes are colored based on the elements of the atoms connected by the bond and its order. Because the four placeholder nodes located at a given atom are likewise identical by symmetry, this adjustment provides for a convenient way to implement the merging of placeholder nodes described in section 1.2.

2 Graph Neural Networks

To help showcase the different convolutional operators, a simple example is provided. Suppose a single-layer GNN-SOM is applied to a three-carbon chain molecule with atoms A-B-C connected in that order. Notice that atoms A and C are symmetrical: they are not distinguishable from one another and as such any calculations performed for A will match those for C and vice versa.
The GIN (Xu et al.) message passing layer uses the following process to generate updated node representations $\mathbf{x}'$ given previous layer representations $\mathbf{x}$:

$$\mathbf{x}_i' = MLP\left((1+\epsilon)\mathbf{x}_i + \sum\nolimits_{j\in N(i)} \mathbf{x}_j\right)$$

where i refers to some node, $N(i)$ is the set of neighbors of node i, ε is a learnable parameter, and MLP is a multilayer perceptron. Our implementation of this operator uses a single-layer MLP, in other words, the above equation can be rewritten as follows, assuming $\mathbf{W}$ is the weight matrix and $\mathbf{b}$ is the bias vector of the MLP:

$$\mathbf{x}_i' = \boldsymbol{W}\left((1+\epsilon)\mathbf{x}_i + \sum\nolimits_{j\in N(i)} \mathbf{x}_j\right) + \boldsymbol{b}$$

The molecular fingerprinting (MF) (Duvenaud et al.) message passing layer uses matrices $\mathbf{W}^{(\deg(i))}$ and $\mathbf{H}^{(\deg(i))}$ as learnable parameters specific to the degree of node i, and the updated node representations $\mathbf{x}'$ given by:

$$\mathbf{x}_i' = \mathbf{W}^{(deg(i))}\mathbf{x}_i + \mathbf{H}^{(deg(i))} \sum\nolimits_{j\,\in\, N(i)} \mathbf{x}_j$$

For each of the atoms in our example, the GIN model would generate the following representations:

$\mathbf{x}'_A = \mathbf{W}((1+\varepsilon)\mathbf{x}_A + \mathbf{x}_B) + \mathbf{b} = (1+\varepsilon)\,\mathbf{W}\mathbf{x}_A + \mathbf{W}\mathbf{x}_B + \mathbf{b}$
$\mathbf{x}'_C = \mathbf{W}((1+\varepsilon)\mathbf{x}_C + \mathbf{x}_B) + \mathbf{b} = (1+\varepsilon)\,\mathbf{W}\mathbf{x}_C + \mathbf{W}\mathbf{x}_B + \mathbf{b}$
$\mathbf{x}'_B = \mathbf{W}((1+\varepsilon)\mathbf{x}_B + \mathbf{x}_A + \mathbf{x}_C) + \mathbf{b} = (1+\varepsilon)\,\mathbf{W}\mathbf{x}_B + \mathbf{W}\mathbf{x}_A + \mathbf{W}\mathbf{x}_C + \mathbf{b}$

The molecular fingerprinting approach would generate superficially similar representations:

$\mathbf{x}'_A = \mathbf{W}^{(1)}\mathbf{x}_A + \mathbf{H}^{(1)}\mathbf{x}_B$
$\mathbf{x}'_C = \mathbf{W}^{(1)}\mathbf{x}_C + \mathbf{H}^{(1)}\mathbf{x}_B$
$\mathbf{x}'_B = \mathbf{W}^{(2)}\mathbf{x}_B + \mathbf{H}^{(2)}(\mathbf{x}_B + \mathbf{x}_C) = \mathbf{W}^{(2)}\mathbf{x}_B + \mathbf{H}^{(2)}\mathbf{x}_B + \mathbf{H}^{(2)}\mathbf{x}_C$

Most notably, the MF layer learns a separate set of parameters for the intermediate atom B. In addition, it uses separate matrices for atom's own features and those of its neighbors. The GIN model, on the other hand, learns one weight matrix for all atoms and only uses a scalar ε to provide differential treatment between an atom and its neighborhood. As a result, given the same depth of GNN, the MF layer can learn more complex functions for predicting SOMs.

The above two methods are examples of spatial GNNs. We also consider the Chebyshev convolutional operator (Defferrard et al.), which is an example of a spectral graph method. Broadly speaking, a GNN layer is a form of a graph filter, a localized operation of graph signals (Zhang et al.), which in case of a GNN are the node features. This filtering can be performed in the vertex domain, where it is commonly defined as a linear combination of signal components in a node i's K-hop neighborhood $N(i, k)$:

$$\mathbf{x}'_i = w_{i,i}\,\mathbf{x}_i + \Sigma_{j\,\in N(i,K)}\,(\mathbf{x}_j \cdot w_{i,j})$$

An equivalent operation can be performed in the spectral domain as well if a K-polynomial is used as a filter signal (Zhang et al.). Such filters are computationally expensive to implement, however, with complexity being quadratic in the number of nodes. The authors of the convolutional operator proposed using a polynomial that can be computed recursively, the Chebyshev expansion, reducing the computational complexity to $O(K\,|E|)$, where E is the set of graph edges. (Defferrard et al.)

The resulting convolution considers features from up to K-hops away from a node at every given layer. Thus, assuming equal number of GNN layers D, the receptive field of a Chebyshev convolution GNN covers a $(D \cdot K)$-hop neighborhood, compared to an only D-neighborhood for conventional spatial GNNs.

References

Defferrard, Michaël et al. "Convolutional Neural Networks on Graphs with Fast Localized Spectral Filtering." 2016, doi:10.48550/ARXIV.1606.09375.

Duvenaud, David et al. "Convolutional Networks on Graphs for Learning Molecular Fingerprints." arXiv, 2015.

Finkelmann, A. R. et al. "Metscore: Site of Metabolism Prediction Beyond Cytochrome P450 Enzymes." *ChemMedChem*, vol. 13, no. 21, 2018, pp. 2281-89, doi:10.1002/cmdc.201800309.

Kanehisa Laboratories. "Kegg Atom Types." https://www.genome.jp/kegg/reaction/KCF.html. Accessed April 4 2022.

Kotera, M. et al. "Kcf-S: Kegg Chemical Function and Substructure for Improved Interpretability and Prediction in Chemical Bioinformatics." *BMC Syst Biol*, vol. 7 Suppl 6, 2013, p. S2, doi:10.1186/1752-0509-7-S6-S2.

McKay, Brendan D. and Adolfo Piperno. "Practical Graph Isomorphism, Ii." *Journal of Symbolic Computation*, vol. 60, 2014, pp. 94-112, doi:https://doi.org/10.1016/j.jsc.2013.09.003.

Rücker, Christoph and Markus Meringer. "How Many Organic Compounds Are Graph–Theoretically Nonplanar?" *MATCH Communications in Mathematical and in Computer Chemistry*, vol. 45, 2002, pp. 153-72.

Sato, Masayuki et al. "Kcf-Convoy: Efficient Python Package to Convert Kegg Chemical Function and Substructure Fingerprints." *bioRxiv*, 2018, p. 452383, doi:10.1101/452383.

Torán, Jacobo and Fabian Wagner. "The Complexity of Planar Graph Isomorphism." 2010.

Xu, Keyulu et al. "How Powerful Are Graph Neural Networks?" arXiv, 2018. doi:10.48550/ARXIV.1810.00826.

Zhang, Si et al. "Graph Convolutional Networks: A Comprehensive Review." *Computational Social Networks*, vol. 6, no. 1, 2019, p. 11, doi:10.1186/s40649-019-0069-y.
